# Real-World Early Treatment with Room Temperature–Stable Recombinant Factor VIIa in Hemophilia A/B and Inhibitors: SMART-7™ Post Hoc Analyses

**DOI:** 10.1055/s-0037-1608943

**Published:** 2017-12-08

**Authors:** Francesco Demartis, Angelika Batorova, Hervé Chambost, Peyman Eshghi, Mehran Karimi, Kaan Kavakli, Soraya Benchikh El Fegoun, Katarina Cepo, Lene Sommer Vestergaard, Gary Benson

**Affiliations:** 1 Agenzia per l'Emofilia, Centro Malattie Emorragiche, Azienda Ospedaliero Universitaria Careggi, Firenze, Italy; 2 National Hemophilia Centre, Department of Hematology and Transfusion Medicine, Medical School of Comenius University, University Hospital, Bratislava, Slovakia; 3 Service d'Hématologie Oncologie Pédiatrique, La Timone, APHM, and Inserm, UMR 1062, Faculté de Médecine, Aix-Marseille Université, Marseille, France; 4 Pediatric Congenital Hematologic Disorders Research Center, Shahid Beheshti University of Medical Sciences, Tehran, Iran; 5 Hematology Research Center, Shiraz University of Medical Sciences, Shiraz, Iran; 6 Department of Hematology, Ege University Children's Hospital, Izmir, Turkey; 7 Biopharm Operations, Novo Nordisk Healthcare AG, Zurich, Switzerland; 8 Medical & Science Biopharm, Novo Nordisk A/S, Søborg, Denmark; 9 Biostatistics LCM Haem, GH & Concizumab, Novo Nordisk A/S, Søborg, Denmark; 10 Northern Ireland Haemophilia Comprehensive Care Centre, Belfast City Hospital, Belfast, United Kingdom

**Keywords:** bleeding, efficacy, eptacog alfa activated, NovoSeven ^®^, rFVIIa

## Abstract

Treating hemophilia A or B patients with inhibitors is particularly challenging, as they do not respond to replacement therapy with factor VIII or factor IX concentrates. A room temperature–stable formulation of recombinant activated factor VII (rFVIIa; NovoSeven ^®^ ), which provides improved convenience and treatment access to patients compared with the earlier formulation of rFVIIa, was shown to be safe and effective in a post-authorization, multinational, observational study (Study Monitoring Antibodies against Room Temperature–stable factor 7 [SMART-7™]). In post hoc, subgroup analyses of SMART-7™ data, the hemostatic response following rFVIIa monotherapy in patients with hemophilia A or B with inhibitors by time to first treatment and in different age cohorts was assessed. A total of 482/618 bleeding episodes treated with rFVIIa monotherapy and with (1) valid efficacy assessment, (2) no missing time for bleed start, (3) no missing time for any dose administration, and (4) valid time to first treatment were included in the analyses. Data on the type and location of bleeding episodes treated with rFVIIa monotherapy were also collected. The majority of bleeding episodes treated with rFVIIa monotherapy were treated within 1 hour after bleeding onset (318/482 [66%]) and, among them, 96.5% (307/318) were effectively treated (i.e., bleeding stopped). Hemostatic efficacy remained high for bleeding episodes treated >1 to ≤4 hours after the onset, with 94/101 (93.1%) treated effectively. Cause and location of bleeding varied across the different age groups assessed. Real-world evidence from post hoc, subgroup analyses of SMART-7™ data confirmed that patients were able to treat themselves quickly and that early treatment with rFVIIa was associated with high efficacy.

## Introduction

Patients with hemophilia A or B and high-titer inhibitors against factor VIII (FVIII) or factor IX (FIX), respectively, do not respond to treatment with FVIII or FIX concentrates. Treatment with agents that bypass the need for FVIII or FIX to initiate hemostasis can be used instead in these patients. Recombinant activated coagulation factor VII (rFVIIa; eptacog alfa activated; NovoSeven ^®^ [Novo Nordisk A/S, Bagsværd, Denmark]) has been used as a bypassing agent in hemophilia A or B patients with inhibitors since its approval in Europe in 1996. 1 rFVIIa bypassing treatment results in hemostasis by binding to the surface of activated platelets, which in turn leads to initiation of factor X (FX) activation and subsequent thrombin generation. 2 The first formulation of rFVIIa required storage at 2 to 8°C. A room temperature–stable formulation of rFVIIa was subsequently developed for the treatment of patients with hemophilia A or B with inhibitors to improve treatment convenience. 3 4 It was approved in Europe in April 2008 5 and in the United States in May 2008. 6 This formulation, which is available at different dose strengths, allows convenient storage and increased portability, without the need for cooling boxes or refrigerators, thus providing patients with improved access to treatment.

The safety and effectiveness of routine use of the commercially available room temperature–stable formulation of rFVIIa in a real-world setting was evaluated in SMART-7™ (Study Monitoring Antibodies against Room Temperature–stable factor 7), a large, post-authorization safety study designed to prospectively monitor patients with hemophilia A or B with inhibitors. In the primary analysis of SMART-7™, no treatment-related safety issues and no FVII binding or neutralizing antibodies were detected. 7 In this observational study, bleeding episodes were predominantly spontaneous and occurred mainly in joints and muscles.

In SMART-7™, patients evaluated the status of bleeding episodes after each treatment and the last evaluation was recorded as the overall bleeding assessment. This is different from previous rFVIIa clinical trials, in which effectiveness was evaluated at specific time points, rather than as an overall assessment. 8 9 We performed a post hoc, subgroup analysis of SMART-7™ data to assess the hemostatic response based on the time to treatment initiation in patients treated with rFVIIa monotherapy. The speed of treatment has a direct impact on therapeutic outcomes in patients with hemophilia and, therefore, assessing the effect of early treatment with rFVIIa monotherapy in a real-world setting is of special interest. 10 We also performed a post hoc subanalysis of SMART-7™ data based on age to explore whether there were any differences in bleeding characteristics or treatment with rFVIIa among different age cohorts. Analyzing the results of the study based on age is of particular importance, as patients with hemophilia have different needs at different stages in their lives. 11 12 13

In this article, results from post hoc subanalyses of the SMART-7™ study assessing hemostatic response with rFVIIa monotherapy by time to first treatment and in different age cohorts are reported.

## Materials and Methods

### Study Design

SMART-7™ was a prospective, observational, single-arm, open-label study, conducted between November 2010 and March 2015 at 24 sites in 14 countries. The primary objective of the study was to prospectively monitor for decreased therapeutic response in patients with hemophilia A or B treated with the room temperature–stable formulation of rFVIIa (NovoSeven ^®^ ) and the development of neutralizing antibodies toward FVII (i.e., FVII inhibitors). As this was an observational study, no medication was dispensed during the study, and all medication used was at the discretion of the treating physician according to the local label and their usual practice, meaning that more than one medication could be administered for the treatment of a single bleeding episode (see section “Results” for more details).

Patients were expected to remain in the study for a maximum of 2 years or until reaching at least 25 exposure days of on-demand treatment with rFVIIa. Any exposure to rFVIIa over the course of one 24-hour period was defined as one exposure day. If two or more doses of rFVIIa were administered on the same day, this was recorded as a single exposure day. If treatment continued beyond 24 hours, each initiated 24-hour period was considered as a separate exposure day. In March 2015, Novo Nordisk and the European Medicines Agency (EMA) agreed to complete the study before all patients had reached 25 exposure days, as the overall number of exposure days exceeded the number stated in the original commitment to the health authorities.

The study was performed in accordance with the Declaration of Helsinki and the Guidelines for Good Pharmacoepidemiology Practices. Approval was obtained from local ethics committees, as required, and all patients and/or their legal guardians provided written informed consent prior to enrolment in the study. The study is registered on ClinicalTrials.gov, with the identifier NCT01220141.

### Patients

Male patients with congenital hemophilia A or B with inhibitors to FVIII or FIX who were treated with the commercially available, room temperature–stable formulation of rFVIIa (NovoSeven ^®^ ) were included in the study. Patients initiating immune tolerance induction were eligible to participate in the study, provided bleeding episodes were treated with NovoSeven ^®^ . Patients had to have adequate venous access at the screening visit. Physicians prescribing NovoSeven ^®^ at participating hemophilia treatment centers identified and enrolled eligible patients. There were no exclusion criteria beyond the contraindications noted in the relevant approved product (either in the European summary of product characteristics or the package label for each participating country).

### Outcome Measures and Assessments

All bleeding episode information, including home therapy with NovoSeven ^®^ , was recorded in a patient diary and transferred to paper case report forms (CRFs) by the investigator. Data were collected at baseline, at optional annual/semiannual post-baseline visits and at the end of the study.

Patients evaluated the status of bleeding episodes after each treatment as “bleed stopped,” “bleed slowed,” or “no change/worsened.” Based on these patient evaluations, treatment was described as “effective,” “partially effective,” or “ineffective,” respectively. Overall bleed outcome was defined as the last given patient evaluation of the bleed. Information on all bleeding episodes, including home treatment, was recorded in a patient diary and transferred to the CRF by the investigator at regular assessment visits. Data on the treatment and nature of the bleeding episodes, including type and location, were collected.

Additional details on the SMART-7™ study design, patients, outcome measures, assessments, and statistical analyses are provided in the study of Kavakli et al. 7

In the post hoc, subgroup analyses presented here, the hemostatic response to treatment with rFVIIa only, that is, with rFVIIa monotherapy, by time to first treatment and in different age cohorts was assessed for bleeds satisfying the following four criteria: (1) valid efficacy assessment, (2) no missing time for bleed start, (3) no missing time for any dose administration, and (4) valid time to first treatment.

### Statistical Analysis

No formal statistical analysis was performed. The descriptive statistical analyses of categorical and numerical variables were based on regulatory-specified age groups. Summaries of categorical variables include frequencies and percentages, and summaries of continuous variables include mean (standard deviation [SD]) and median (range) values.

## Results

### Data Sets for the SMART-7™ Primary and Post Hoc Analyses

The primary safety analysis dataset, results from which were recently published, comprised all 51 patients who enrolled in SMART-7™. 7 Forty-eight of the 51 enrolled patients reported having bleeding episodes during the study, amounting to a total of 618 bleeding episodes. Of these, nine episodes did not have an efficacy evaluation, amounting to a total of 609 evaluable bleeding episodes. At the end of treatment, 569 of 609 (93.4%) bleeding episodes with an efficacy evaluation had resolved; 35 of 609 (5.7%) had slowed; and 5 of 609 (0.8%) were unchanged/had worsened. 7 rFVIIa (NovoSeven ^®^ ) was the main hemostatic agent used in SMART-7™, and was used to treat 591 of 618 (95.6%) bleeding episodes. Other hemostatic medications used, either individually or for the same bleed as rFVIIa, included plasma-derived activated prothrombin complex concentrate (pd-aPCC; FEIBA) in 64 of 618 (10.4%) bleeding episodes, the previous formulation of rFVIIa in 1 of 618 (0.2%) episodes, and medications categorized as “other” in 6 of 618 (1.0%) episodes. The “other” medications included plasma-derived FVIII, rFVIII (Helixate NexGen), and rFIX (BeneFix). Concomitant antifibrinolytics were used in the treatment of 62 of 618 (10%) bleeding episodes.

**Fig. 1:**
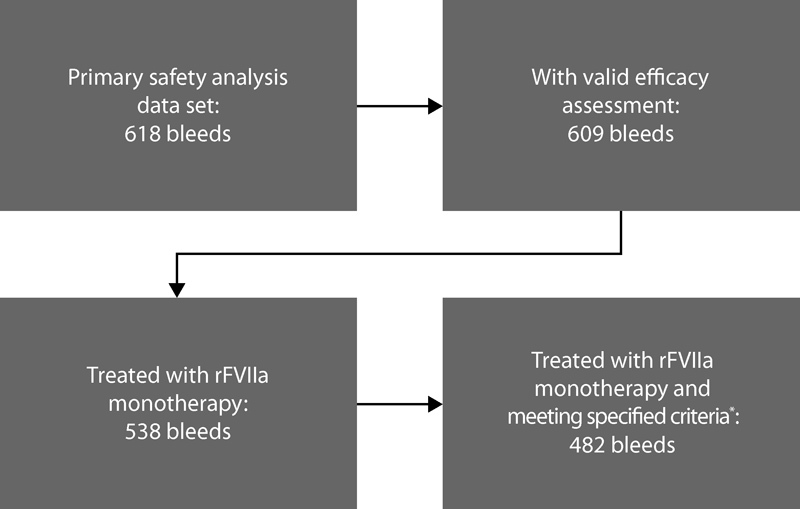
SMART-7™ primary and post hoc subgroup analysis datasets. The primary safety analysis dataset includes 609 bleeding episodes with valid efficacy assessment. 7 *The rFVIIa monotherapy dataset includes 482 bleeding episodes with (1) valid efficacy assessment, (2) no missing time for bleed start, (3) no missing time for any dose administration, and (4) valid time to first treatment.

A total of 538 bleeding episodes were treated with rFVIIa monotherapy during SMART-7™. Among these, 56 bleeding episodes did not meet the four criteria specified in the post hoc analysis (i.e., valid efficacy assessment, no missing time for bleed start, no missing time for any dose administration, and valid time to first treatment). Therefore, a total of 482 bleeding episodes (reported by 45 patients) were included in the rFVIIa monotherapy post hoc analysis dataset that is discussed herein ( Fig. 1 ).

### Age Demographics of Patients Treated with rFVIIa Monotherapy

Among the 45 patients with bleeding episodes that were treated with rFVIIa monotherapy and who met the four criteria specified in the subanalysis, the mean (SD) age was 28.1 (21.2) years. The majority of the patients (25/45 [55.6%]) were ≥18 to ≤65 years old. The second largest age category comprised patients aged ≥2 to <6 years (9/45 [20.0%]).

### Characteristics of Bleeding Episode in Different Age Cohorts in Patients Treated with rFVIIa Monotherapy

Overall, the majority (316/482 [65.6%]) of bleeding episodes treated with rFVIIa monotherapy were spontaneous ( Table 1 ). However, the proportion of spontaneous versus traumatic bleeds differed among the different age groups that were assessed. More specifically, adolescent patients (≥12 to <18 years) experienced the highest proportion of spontaneous bleeds (96.2%) and the lowest proportion of traumatic bleeds (3.8%). The highest proportion of traumatic bleeds (63.6%) was reported in children aged ≥2 to <6 years.

**Table 1:** Characteristics of bleeding episodes treated with rFVIIa (NovoSeven ^®^ ) monotherapy in SMART-7™ overall and by age group

Age (y)	<2	≥2 to <6	≥6 to <12	≥12 to <18	≥18 to ≤65	>65	All
Patients ( *n* )	2	9	4	3	25	2	45
**Cause of bleed,** ***n*** **(%)**							
* N* (%)	16 (100)	77 (100)	45 (100)	53 (100)	262 (100)	29 (100)	482 (100)
Spontaneous	9 (56.3)	25 (32.5)	8 (17.8)	51 (96.2)	203 (77.5)	20 (69.0)	316 (65.6)
Traumatic	7 (43.8)	49 (63.6)	26 (57.8)	2 (3.8)	49 (18.7)	8 (27.6)	141 (29.3)
Other a	Not reported	3 (3.9)	11 (24.4)	Not reported	10 (3.8)	1 (3.4)	25 (5.2)
**Location of bleed,** ***n*** **(%)** b							
* N* (%)	16 (100)	81 (100)	49 (100)	54 (100)	273 (100)	30 (100)	503 (100)
Head	Not reported	4 (4.9)	Not reported	2 (3.7)	1 (0.4)	Not reported	7 (1.4)
Mouth/gums	3 (18.8)	7 (8.6)	2 (4.1)	Not reported	7 (2.6)	5 (16.7)	24 (4.8)
Muscle	4 (25.0)	24 (29.6)	13 (26.5)	18 (33.3)	42 (15.4)	9 (30.0)	110 (21.9)
Target joint	Not reported	10 (12.3)	13 (26.5)	12 (22.2)	143 (52.4)	12 (40.0)	190 (37.8)
Nontarget joint	1 (6.3)	19 (23.5)	8 (16.3)	17 (31.5)	50 (18.3)	Not reported	95 (18.9)
Skin	1 (6.3)	6 (7.4)	1 (2.0)	3 (5.6)	4 (1.5)	1 (3.3)	16 (3.2)
Other c	7 (43.8)	11 (13.6)	12 (24.5)	2 (3.7)	26 (9.5)	3 (10.0)	61 (12.1)

Notes: Bleeds treated with recombinant factor VIIa (rFVIIa) monotherapy and with (1) valid efficacy assessment, (2) no missing time for bleed start, (3) no missing time for any dose administration, and (4) valid time to first treatment were included.

a “Other” bleed causes were as follows: swimming, pace maker removal, strain, light physical activity, household activities, blood sampling, subcutaneous hemorrhage, throat bleed, portacath protruding through skin, accidental injury, suspected trauma (not confirmed), dehiscence of wound, re-establishment of previous bleeding, vomiting, pressure, trauma and beating, and unknown.

b A bleeding episode could have a bleed in more than one location.

c “Other” bleed locations were as follows: urine, forearm, prior portacath scar, lip, brain (frontal hematoma), ear (mastoiditis), hip, skin, forehead, postoperative central venous device site, kidney, nose (epistaxis), head trauma, wrist, conjunctiva, toe, neck, shoulder, subcutaneous, soft tissue, abdominal, thumb, throat, hand, genital area, foot, thigh, leg, knee, vomit (hematemesis), feces (melena), right side of body, port site, back, buttock, wound, venepuncture site, finger, palm, stomach mucous membrane (not confirmed), ankle, tongue, pelvis, and unknown.

Overall, target joints were the most common bleed location (37.8%). Similar to bleed cause, bleed location also varied across the different age groups assessed ( Table 1 ). For children aged ≥2 to <6 years and adolescents aged ≥12 to <18 years, the greatest proportion of bleeds occurred in muscles (29.6 and 33.3%, respectively). In adults aged ≥18 to ≤65 years and >65 years, the majority of bleeding episodes occurred in target joints (52.4 and 40.0%, respectively). Conversely, in infants <2 years old, no bleeds were reported in target joints.

### Hemostatic Response with rFVIIa Monotherapy Overall and by Time to First Treatment

At the time of diary completion, 456 of 482 (94.6%) bleeding episodes treated with rFVIIa monotherapy had resolved, 23 of 482 (4.8%) had slowed, and 3 of 482 (0.6%) remained unchanged/had worsened. The three bleeding episodes that were reported as “unchanged/worsened” occurred in three separate patients. One of these patients experienced three bleeds before and two bleeds after the ineffective treatment, all of which resolved. The second and third patients each experienced one bleed that resolved prior to the treatment reported as ineffective. The third patient ultimately died following a physical assault and the development of sepsis not related to rFVIIa.

**Fig. 2:**
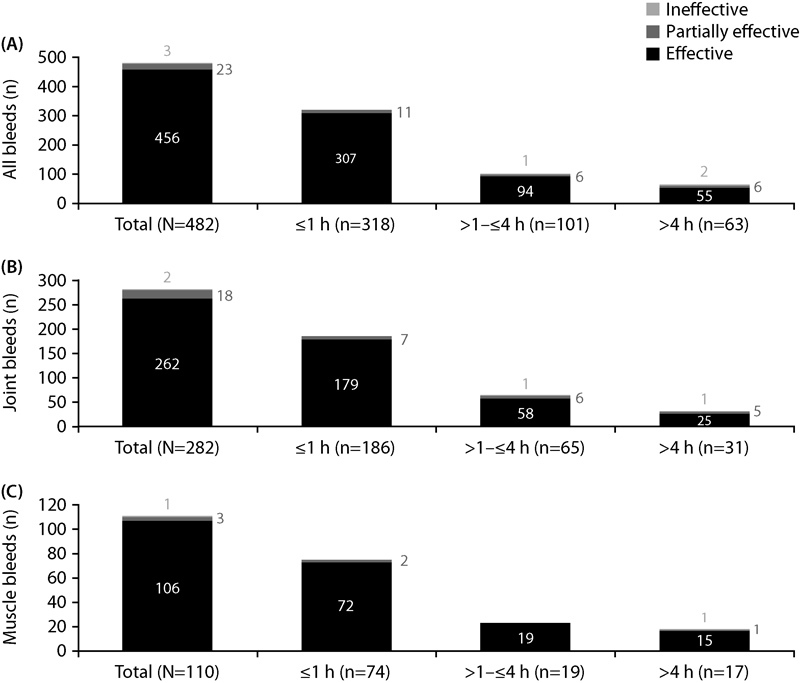
Hemostatic response with rFVIIa (NovoSeven ^®^ ) monotherapy by time to first treatment in patients with hemophilia A or B and inhibitors in SMART-7™. Bleeding episodes treated with recombinant factor VIIa (rFVIIa) monotherapy and with (1) valid efficacy assessment, (2) no missing time for bleed start, (3) no missing time for any dose administration, and (4) valid time to first treatment were included.

The median (min; max) time from bleed start to first dose for the 482 bleeding episodes treated with rFVIIa monotherapy in SMART-7™ was 30 minutes (0.0; 10,335), indicating that the majority of bleeds were treated quickly. Rapid treatment following bleed onset was confirmed, as the majority of bleeding episodes treated with rFVIIa monotherapy (318/482 [66%]) was treated within 1 hour. Among the 318 bleeding episodes that were treated with rFVIIa monotherapy within 1 hour after bleed start, 307 (96.5%) were treated effectively ( Fig. 2 ). Partially effective treatment was reported for 11 of 318 (3.5%) bleeds treated within 1 hour from onset of bleeding. A total of 63 of 482 bleeds, representing only 13.1% of bleeds overall, were treated >4 hours after bleed onset, with 55 of 63 (87.3%) being treated effectively. Early treatment (i.e., ≤1 hour) with rFVIIa monotherapy was effective for both joint and muscle bleeds, with 179 of 186 (96.2%) and 72 of 74 (97.3%) bleeds resolving, respectively ( Fig. 2 ). The majority of bleeding episodes treated with rFVIIa monotherapy ≤1 hour from onset were spontaneous (68.6%) and occurred in target joints (38.4%; Table 2 ).

**Table 2:** Characteristics of bleeding episodes treated ≤ 1 hour from bleed onset with rFVIIa (NovoSeven ^®^ ) monotherapy in SMART-7™ overall and by age group

Age (y)	<2	≥2 to <6	≥6 to <12	≥12 to <18	≥18 to ≤65	>65	All
Patients ( *n* )	2	8	4	3	23	2	42
**Cause of bleed,** ***n*** **(%)**							
* N* (%)	7 (100)	36 (100)	39 (100)	51 (100)	158 (100)	27 (100)	318 (100)
Spontaneous	5 (71.4)	7 (19.4)	6 (15.4)	49 (96.1)	133 (84.2)	18 (66.7)	218 (68.6)
Traumatic	2 (28.6)	29 (80.6)	22 (56.4)	2 (3.9)	20 (12.7)	8 (29.6)	83 (26.1)
Other	Not reported	Not reported	11 (28.2)	Not reported	5 (3.2)	1 (3.7)	17 (5.3)
**Location of bleed,** ***n*** **(%)** a							
* N* (%)	7 (100)	39 (100)	43 (100)	52 (100)	162 (100)	28 (100)	331 (100)
Head	Not reported	3 (7.7)	Not reported	2 (3.8)	Not reported	Not reported	5 (1.5)
Mouth/gums	2 (28.6)	3 (7.7)	2 (4.7)	Not reported	4 (2.5)	4 (14.3)	15 (4.5)
Muscle	1 (14.3)	12 (30.8)	13 (30.2)	18 (34.6)	21 (13.0)	9 (32.1)	74 (22.4)
Target joint	Not reported	4 (10.3)	10 (23.3)	10 (19.2)	92 (56.8)	11 (39.3)	127 (38.4)
Nontarget joint	Not reported	10 (25.6)	7 (16.3)	17 (32.7)	27 (16.7)	Not reported	61 (18.4)
Skin	1 (14.3)	3 (7.7)	1 (2.3)	3 (5.8)	3 (1.9)	1 (3.6)	12 (3.6)
Other	3 (42.9)	4 (10.3)	10 (23.3)	2 (3.8)	15 (9.3)	3 (10.7)	37 (11.2)

Notes: Bleeds treated with recombinant factor VIIa (rFVIIa) monotherapy and with (1) valid efficacy assessment, (2) no missing time for bleed start, (3) no missing time for any dose administration, and (4) valid time to first treatment were included. *N* : number of bleeds with time to treatment ≤ 60 minutes.

a A bleeding episode could have a bleed in more than one location.

The median total dose of rFVIIa used among the monotherapy subgroup of patients was 277.8 μg/kg (interquartile range [Q3–Q1; IQR]: 397.0 μg/kg). The total dose of rFVIIa administered was comparable for bleeds treated ≤1 hour and those treated >1 to ≤4 hours or >4 hours after bleed onset. The median total dose (IQR) was 279.5 (412.7), 272.7 (327.6), and 280.2 (574.6) μg/kg for bleeds treated ≤1 hour, >1 to ≤4 hours, and >4 hours after bleed onset, respectively. The total dose (IQR) of rFVIIa administered were also assessed by bleed location, with 280.1 (495.5), 277.0 (350.3), and 275.4 (437.8) μg/kg for muscle, target joint, and nontarget joint bleeds, respectively.

### Hemostatic Response in Different Age Cohorts and by Time to Treatment with rFVIIa Monotherapy

**Fig. 3:**
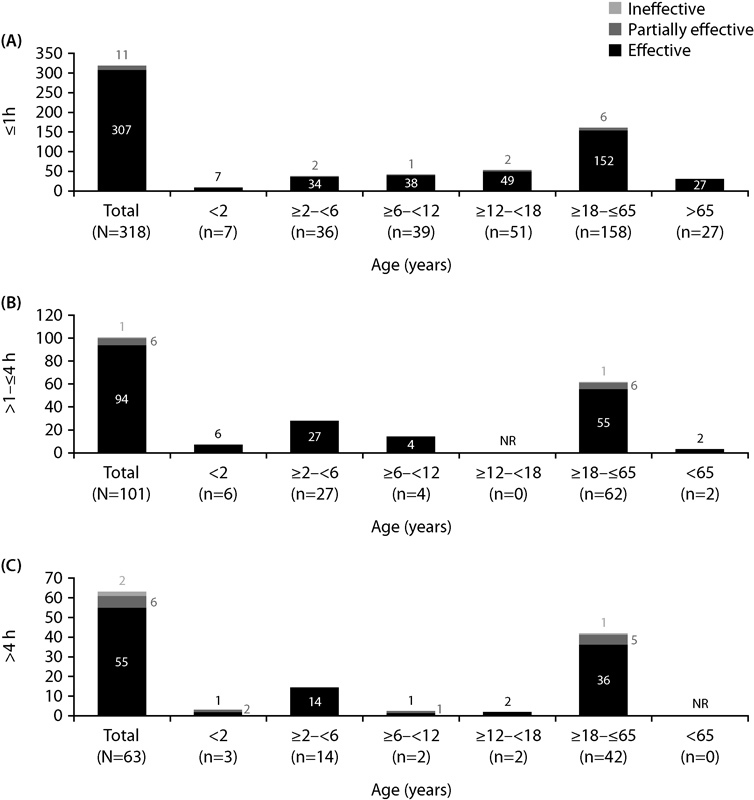
Hemostatic response with rFVIIa (NovoSeven ^®^ ) monotherapy by age and time to treatment in SMART-7™. Bleeding episodes treated with recombinant factor VIIa monotherapy and with (1) valid efficacy assessment, (2) no missing time for bleed start, (3) no missing time for any dose administration, and (4) valid time to first treatment were included. NR, not reported.

The largest number of bleeds was observed among patients who were ≥18 to ≤65 years old ( Fig. 3 ). It should be noted that this was also the age group with the largest number of patients. There did not appear to be any differences in hemostatic effectiveness with rFVIIa monotherapy for bleeds treated within 1 hour in any of the age groups assessed. Data in this subgroup analysis confirmed that the majority of patients were able to treat their bleeds early and according to the label.

## Discussion

rFVIIa has consistently shown high efficacy rates in data collected by international post-approval registries established to assess its effectiveness and safety, such as the U.S.-based Hemostasis and Thrombosis Research Society (HTRS) registry, 14 the international ONE registry, 15 and the Czech HemoRec registry. 16 17 In SMART-7™, a large, prospective, postmarketing study in patients with congenital hemophilia with inhibitors, no treatment-related safety issues and no FVII binding antibodies were reported for patients treated with the room temperature–stable formulation of rFVIIa (NovoSeven ^®^ ), while high hemostatic effectiveness was observed, with 93.4% of all treatment-requiring bleeding episodes stopping following treatment. 7 We performed post hoc subanalyses of data collected during SMART-7™ to assess the hemostatic response to rFVIIa monotherapy based on time to treatment following bleed onset as well as in different age cohorts.

We observed a high hemostatic response of 94.6% for bleeding episodes treated with rFVIIa monotherapy (and satisfying the four constraints specified in the analysis, as previously described). These real-world efficacy data correspond well with the response rates reported in rFVIIa randomized clinical trials in hemophilia patients with inhibitors experiencing mild-to-moderate bleeding episodes. 18 19 20 21

Rapid bleeding control is critical in patients with hemophilia. The World Federation of Hemophilia (WFH) clinical guidelines recommend that patients with hemophilia are treated early (within 2 hours of onset for acute bleeds) to ensure fast pain relief and to limit long-term disability and hospitalization. 10 In hemophilia patients with inhibitors in particular, rapid treatment is associated with faster bleeding resolution and fewer administrations of therapeutic product compared with later treatment. 22 23 Early treatment also limits joint and musculoskeletal complications, the severity of which is proportional to the amount of blood deposited in the joint, as well as the duration of joint exposure to blood. 24 The room temperature–stable formulation of rFVIIa is conducive to early treatment, as it is easy to reconstitute, with a fast injection time. In SMART-7™, the majority of patients were able to treat themselves early (<1 hour from onset of bleed), resulting in very high hemostatic effectiveness (96.5%) among those who received rFVIIa monotherapy. Hemostatic effectiveness for bleeds treated <1 hour from onset of bleed was very similar to the overall effectiveness observed with rFVIIa monotherapy (96.5 vs. 94.6%). Effectiveness remained high (87.3%) for bleeding episodes that were treated late (>4 hours after onset of bleed), although these episodes represented just over 13% of the reported bleeds overall. Moreover, early treatment (≤1 hour) with rFVIIa monotherapy was effective for both joint and muscle bleeds. Results from this subgroup analysis, therefore, indicate that hemostatic effectiveness with rFVIIa can be expected to be highest when treatment is initiated early (i.e., within 1 hour from bleed onset), while efficacy can still be expected to be high for bleeds treated as late as 4 hours after onset of bleeding. These results are in line with those reported in the Czech HemoRec registry, which also showed a real-world benefit with early rFVIIa treatment. 17 Patients included in that study who were treated with rFVIIa ≤2 hours after bleeding onset experienced less re-bleeding compared with patients who were treated >2 hours after onset (5.2 vs. 13.7%, respectively).

rFVIIa consumption was comparable across patients treated with monotherapy at different time points in SMART-7™. The initial median dose and the total dose administered were comparable for bleeds treated ≤1 hour and those treated >1 to ≤4 hours or >4 hours after bleed onset, indicating that early treatment with rFVIIa can lead to high effectiveness without requiring increased dosing. Similar to other real-world data, such as those from the ONE registry, the HemoRec registry, and the Dosing Observational Study in Hemophilia (DOSE), there was variability in the initial dosing patterns followed by patients, with some using nonstandard doses. 15 17 25 Real-world data have shown that rFVIIa dosing is often individualized to provide the optimal treatment outcome in each case. For example, in DOSE, it was reported that higher initial rFVIIa doses than those prescribed were actually administered for joint bleeds (median: 231.3 vs. 123.0 μg/kg in adults), 25 while in the ONE registry, 26% of bleed episodes fell within an “intermediate” initial dose category (>120 to <250 μg/kg). 15 The variability in initial dosing did not seem to have an effect on the overall hemostatic effectiveness with rFVIIa in SMART-7™. The initial and total doses of rFVIIa administered were also comparable for muscle, target joint, and nontarget joint bleeds.

In this post hoc analysis, we also assessed characteristics of bleeding episode among different age cohorts in patients treated with rFVIIa monotherapy in SMART-7™. Although the number of patients in each age cohort was small (over half of the patients were aged ≥18 to ≤65 years), meaning that a conclusive statement cannot be made, the data suggest that there was heterogeneity among patients of different ages with regard to the cause and location of bleeding. Overall, the majority of bleeding episodes were spontaneous and occurred in joints.

Given the observational nature of SMART-7™, there is the potential of selection bias. The small number of patients in each of the age cohorts assessed and the differences between the time-to-treatment subgroup sizes are limitations of the study that should be taken into account. It is also possible that the rFVIIa monotherapy group could have largely comprised patients with milder disease or bleeding, as these patients may be more likely to receive a single effective treatment compared with patients with more severe bleeding. In addition, the results need to be considered in light of the fact that this was a post hoc analysis. As data collection was based on patient-reported diaries, there is a possibility that missing data (e.g., information on the start or stop times of a bleeding episode or treatment) may have had an impact on the results and interpretation of this post hoc analysis.

In conclusion, results from post hoc, subgroup analyses of SMART-7™ data confirmed the importance of early treatment in hemophilia A or B patients with inhibitors for all bleeds. The results with rFVIIa early treatment were also comparable among all age groups assessed.
